# Urinary polycyclic aromatic hydrocarbon excretion and regional body fat distribution: evidence from the U.S. National Health and Nutrition Examination Survey 2001–2016

**DOI:** 10.1186/s12940-022-00890-8

**Published:** 2022-08-10

**Authors:** Yeli Wang, Lu Zhu, Tamarra James-Todd, Qi Sun

**Affiliations:** 1 Department of Nutrition, Harvard T.H. Chan School of Public Health, 677 Huntington Avenue, Boston, MA 02115 USA; 2 Department of Environmental Health, Harvard T.H. Chan School of Public Health, 655 Huntington Avenue, Boston, MA 02115 USA; 3 Department of Epidemiology, Harvard T.H. Chan School of Public Health, 677 Huntington Avenue, Boston, MA 02115 USA; 4 Channing Division of Network Medicine, Department of Medicine, Brigham and Women’s Hospital and Harvard Medical School, 181 Longwood Avenue, Boston, MA 02115 USA

**Keywords:** Polycyclic aromatic hydrocarbons, non-smoker, racial/ethnic difference, obesity, body fat distribution, NHANES

## Abstract

**Background:**

Polycyclic aromatic hydrocarbons (PAHs) are environmental pollutants that may contribute to the etiology of obesity. However, it is unclear whether PAHs from environmental sources are associated with regional body fat distribution, and whether the association varies across racial/ethnic groups who may have differential PAH exposure patterns.

**Objectives:**

To examine correlations between PAHs and body fat distribution, and potential racial/ethnic differences among U.S. adults.

**Methods:**

Ten PAHs were measured in spot urine samples from 2691 non-smoking adults (age ≥ 20 years) in the NHANES 2001–2016. Dual-energy X-ray absorptiometry was used to measure fat mass percent (FM%). Partial Pearson correlation coefficients (*r*) with multivariable adjustment were used to assess PAH-FM% associations.

**Results:**

In the total population, 1-naphthalene, 3-fluorene, and 1-pyrene were inversely correlated with total FM% or trunk FM% (adjusted *r* ranged: − 0.06 to − 0.08), while 2-naphthalene, 9-fluorene, and 4-phenanthrene were positively correlated with the FM% measurements (*r*: 0.07–0.11). PAH levels are highest among non-Hispanic Blacks, followed by Hispanics and Whites and some of the correlations were different by these races/ethnicities. Among non-Hispanic Whites, no PAH was correlated with FM%. In contrast, 9-fluorene was positively correlated with total FM% (*r* = 0.20) and trunk FM% (*r* = 0.22) among Blacks, and 4-phenanthrene was positively correlated with total FM% (*r* = 0.23) and trunk FM% (*r* = 0.24) among Hispanics (*P*-interaction: 0.010–0.025).

**Discussion:**

In this US adult population, certain PAHs are significantly associated with higher body fat contents among non-Hispanic Blacks and Hispanics but not non-Hispanic Whites, suggesting that minority groups might be particularly susceptible to PAH’s obesogenic effects or the effects of other factors that determine the PAH exposure levels. Alternatively, differences in body composition may contribute to differential PAH metabolism in minority groups. Future studies are warranted to explore the racial/ethnic disparity in PAH exposures, drivers of these exposure differences, and mechanisms through which PAHs may influence body composition by races/ethnicities.

**Supplementary Information:**

The online version contains supplementary material available at 10.1186/s12940-022-00890-8.

## Introduction

Polycyclic aromatic hydrocarbons (PAHs) are environmental pollutants that may exert detrimental effects on human health [1]. PAHs are generated through incomplete combustion of organic materials (e.g., coal, oil, gas, wood, garbage, and tobacco) [2]. Common nonoccupational routes of PAH exposures include inhalation (e.g., air contaminated with motor vehicle exhaust, cigarette smoke, wood smoke), diet (e.g., grilled/charred meats or foods, contaminated flour and bread products, processed and pickled foods, and contaminated water and cow’s milk), and skin absorption (e.g., soil and dust) [2, 3]. After absorption, PAHs are transported by chylomicrons and stored mostly in adipose tissue, kidney, and liver [3, 4].

PAHs are potential carcinogens [2], and emerging evidence has suggested that exposure to PAHs may also contribute to the development of metabolic disorders, including diabetes [5, 6], dyslipidemia [6], hypertension [6, 7], and cardiovascular disease [7]. Hydroxylated PAHs, the main metabolic products of PAHs, are endocrine disruptors that mimic estrogens and inhibit thyroid and androgen functions [8–11]. In an animal study, PAH exposure impaired adipose tissue lipolysis and resulted in increased body weight and fat mass in mice [12]. Epidemiological studies among children and adults also showed that urinary excretion of PAHs was positively associated with higher levels of body mass index (BMI), waist circumference (WC), or waist-to-height ratio [6, 13–17]. However, evidence is limited regarding the associations between PAH exposures and body fat distribution in adults.

Body fat distribution has been recognized to play a significant role in the etiology of metabolic diseases [18]. There is mounting evidence that the biological functions of adipose tissue may differ by locations [18–20]. To the best of our knowledge, no study has assessed the associations between individual PAHs and various fat depots (e.g., trunk fat, leg fat, lean mass). In addition, it is known that fat deposition and body fat distribution may differ by race/ethnicity [21]. A recent study conducted among U.S. non-smokers observed the highest levels of urinary PAHs among non-Hispanic Blacks [22], highlighting an environmental exposure disparity in PAHs that could contribute to possible differences in related health outcomes. However, given these exposure differences, no study has examined whether the associations between PAHs and body fatness vary by racial/ethnic groups.

To fill these knowledge gaps, we examined the direction and strength of correlations of urinary PAHs with different body anthropometric measurements, including fat mass (total fat, trunk fat, leg fat), lean mass (bone mineral density, total lean mass), BMI, and WC among a large sample of U.S. adults from the National Health and Nutrition Examination Survey (NHANES) 2001–2016. We explicitly compared correlations of PAHs among major racial/ethnic groups (non-Hispanic Whites, non-Hispanic Blacks, and Hispanics) and hypothesized that PAHs are differentially associated with body fat distribution by race/ethnicity.

## Methods

### Study population

The NHANES is a nation-wide representative survey assessing the health and nutritional status of residents in the United States. The detailed study design has been described previously [23]. Briefly, for each biennial NHANES survey a complex sampling process was used to randomly select U.S. residents who are representative of the civilian non-institutionalized population. Participants completed interviews at their homes with trained health professionals and underwent physical examinations in a mobile examination center. During the physical examination, a single spot urine sample was collected from NHANES participants aged six years and older. Written informed consents were obtained from all participants. The study protocol was approved by the institutional review board at the Centers for Disease Control and Prevention (Atlanta, Georgia) [23].

The current study combined data from six NHANES cycles (2001–2002, 2003–2004, 2005–2006, 2011–2012, 2013–2014, 2015–2016) among participants ≥20 years old. We excluded two cycles (2007–2008, 2009–2010) because the measurements on body fat distribution were unavailable. Since cigarette smoking is a predominant exposure source of PAHs among smokers, and smoking is known to modulate body weight [24], the association between PAHs and body fat distribution can be largely driven by smoking status. To avoid the strong confounding of cigarette smoking on the associations between PAHs and body fat contents, we restricted the current analysis to non-smokers. In addition, participants below 20 years old (*n* = 27,842), without medical examination data (*n* = 2284) or complete DXA scan (*n* = 10,214), with medical conditions that may influence body fat distribution (kidney disease, physical limitations, diabetes, cardiovascular disease, asthma, pulmonary disease, and cancer) (*n* = 2504), and with missing values in smoking status (*n* = 3) were excluded. Of the 61,049 surveyed residents, 7531 individuals had measurements of both PAH levels and body fat distribution. Of them, 2691 non-smokers were included in the main analysis after exclusions. The flowchart of the study design is shown in Supplemental Fig. S1.

### Measurements of urinary PAH metabolites

In NHANES study, urinary monohydroxylated metabolites of PAHs were measured in approximately one-third subsample of all participants 6 years and older in the six NHANES cycles. Ten urinary PAHs were measured, including two naphthalene metabolites (1-napthalene, 2-napthalene), three fluorene metabolites (2-fluorene, 3-fluorene, 9-fluorene), four phenanthrene metabolites (1-phenanthrene, 2-phenanthrene, 3-phenanthrene, 4-phenanthrene), and one pyrene metabolite (1-pyrene). In addition to the ten individual PAHs, we created four other variables: the sum of the molar mass of all PAH metabolites, total naphthalene metabolites, total phenanthrene metabolites, and total phenanthrene metabolites, respectively. Of note, the number of PAHs measured in each NHANES cycle varied: it was nine in 2001–2002, ten in 2003–2004/2005–2006/2011–2012, and six in 2013–2014/2015–2016 cycles. We included PAHs measured in at least two survey cycles to ensure a reasonable sample size.

The detailed procedure of laboratory measurement has been described previously [25]. In brief, PAHs were measured in the single spot urine sample by capillary gas chromatography and high-resolution mass spectrometry. To minimize the variability of urinary PAH levels due to differential dilutions, creatinine-adjusted PAHs (dividing PAH concentrations [nanograms per liter urine] by creatinine concentrations [grams per liter urine]) were used in the current analysis as recommended previously [26]. Most PAHs had a detection rate of > 99%, and the detection rate for 4-phenanthrene and 9-fluorene was 89 and 93%, respectively. The coefficient of variation (CV) of PAH measurements ranged from 5 to 13%.

### Dual-energy X-ray absorptiometry (DXA) measurements

DXA scans were administered to eligible survey participants 8 years of age and older in the NHANES mobile examination centers. Females with a positive pregnancy test or those who reported being pregnant at the time of the exam were excluded from the DXA examination. Individuals who reported taking tests with radiographic contrast material in the past 72 hours, participants in nuclear medicine studies in the past 3 days, or those who had a self-reported weight (> 300 pounds) or height (> 6′5″) over the DXA table limit were also excluded from the DXA examination [27]. The whole body DXA was taken with a Hologic QDR-4500A fan-beam densitometer (Hologic, Inc., Bedford, Massachusetts) and provided fat mass and lean mass measurements for the total body, both arms, both legs, the trunk, and the head. Hologic Discovery software was used to derive fat mass and lean mass including total fat, trunk fat, leg fat, bone mineral density, and total lean mass. Multiple imputations were conducted to impute missing data for attenuating any potential biases due to missing DXA data. Specifically, missing readings for DXA data were imputed five times by the sequential regression multivariate imputation [27]. Fat mass percentage (FM%) for total body, trunk and legs were calculated as: FM% = fat mass (kg)/total mass (kg) × 100%.

### Measurements of body mass index and waist circumference

Body measurements such as weight (kg), height (cm), and WC (cm) were measured by trained health professionals following standardized protocols [28]. BMI was calculated as weight divided by height squared (kg/m^2^).

### Covariates

Information on demographics, lifestyle, medical history, and dietary intake was collected using survey questionnaires during the face-to-face interview [29]. Demographic information included age (years), gender (men, women), race/ethnicity (non-Hispanic White, non-Hispanic Black, Hispanic, or others), education (high school or below, any college, and college graduate or above), and poverty income ratio (< and ≥ 1). The poverty income ratio was used as an indicator of socioeconomic status and was calculated by dividing family income by the poverty threshold adjusted for family size and inflation. Lifestyle factors consisted of alcohol use (nondrinkers, 1–3 drinks/day, and ≥ 4 drinks/day) and moderate-to-vigorous physical activity (yes, no). Dietary intake included total calorie intake (kcal/day) and protein intake (gram/day). Serum cotinine levels (ng/mL), an indicator for environmental tobacco smoke among non-smokers [30], were measured by the isotope-dilution high-performance liquid chromatography/atmospheric pressure chemical ionization tandem mass spectrometric method with a lower detection limit of 0.015 ng/ml. C-reactive protein (CRP) levels were quantified by latex-enhanced nephelometry, with a lower detection limit of 0.1 mg/L.

### Statistical analysis

Since the number of missing values varies among PAHs, sample size may vary for individual PAHs. Log-transformation (e base) was applied for creatinine-adjusted PAHs and FM% (total fat, trunk fat, leg fat, trunk/leg ratio), bone mineral density, total lean mass, BMI, and WC to improve the normality. In this cross-sectional analysis, we calculated partial Pearson correlation coefficients (*r*) weighted by the NHANES sampling weight with adjustment of potential confounding factors to examine correlations between log-transformed PAHs and FM%, bone mineral density, total lean mass, BMI, and WC among non-smokers. Covariates adjusted included age, gender, race/ethnicity, education, poverty income ratio, moderate-to-vigorous physical activity, alcohol use, total calorie intake, protein intake, and serum cotinine and CRP levels. Missing values of continuous covariates were replaced with median values. For categorical variables, a missing indicator variable was created for missing values. To examine the potential differences by race/ethnicity, we performed stratified analyses by main racial/ethnic groups (non-Hispanic White, non-Hispanic Black, and Hispanic). Correlation coefficients of creatinine-adjusted PAHs with FM% were transformed to Fisher *z* scores and compared using the Student’s t-test between two groups. We also compared correlations of PAHs with trunk FM% versus leg FM% using the Wolfe’s method [31, 32]. Due to the multiple-imputation procedure, each participant has five sets of measured and imputed values of body fat; correlations were calculated within each DXA dataset, and combined into a single composite estimate using the method of Rubin and Schenker [33].

To account for multiple comparisons, False Discovery Rate (FDR) Benjamini-Hochberg Procedure was used for the correction of *P* values. Statistical significance was determined by a two-sided FDR *P* value smaller than 0.05. All data were analyzed in RStudio version 1.4.1106 (Rstudio, PBC) and SAS version 9.4 (SAS Institute, Cary, NC).

## Results

Characteristics of non-smoking adults stratified by race/ethnicity are shown in Table 1. In the total population, the median age was 37.9 years (interquartile range: 28.0–48.2) and 47.4% were men. In addition, 35.6% were non-Hispanic Whites, 21.7% were non-Hispanic Blacks, and 28.5% were Hispanics. Compared with non-Hispanic Whites, non-Hispanic Blacks and Hispanics were younger and had lower education and lower income. In addition, non-Hispanic Blacks had the highest urinary levels for all ten PAHs compared to non-Hispanic whites and Hispanics. The levels of three out of ten PAHs (i.e., 2-naphthalene, 2-fluorene, and 1-pyrene) were higher among Hispanics compared to non-Hispanic Whites; the remaining seven PAHs had similar levels between non-Hispanic Whites and Hispanics. Non-Hispanic Blacks and Hispanics had higher BMI and leg FM% than non-Hispanic Whites. In addition, Hispanics had higher total FM% and trunk FM% and lower total lean mass compared to non-Hispanic Whites and non-Hispanic Blacks (Table 1).

**Table 1:** Characteristics of study population among non-smokers aged 20 years stratified by race/ethnicity

Variables^**a**^	Non-smokers			
	Total	Non-Hispanic White	Non-Hispanic Black	Hispanic
**Number of participants**^**b**^	2691 (100.0%)	959 (35.6%)	585 (21.7%)	767 (28.5%)
**Age (years)** median (interquartile)	37.9 (28.0, 48.2)	40.4 (29.7, 49.8)	35.9 (26.6, 45.6)	33.1 (25.5, 41.8)
**Gender, men**	1221 (47.4%)	471 (49.1%)	273 (46.7%)	310 (40.4%)
**Education**				
**High school and below**	1042 (30.6%)	259 (27.0%)	214 (36.6%)	495 (64.5%)
**Any college**	816 (31.6%)	311 (32.4%)	225 (38.5%)	181 (23.6%)
**College graduate and above**	833 (37.8%)	389 (40.6%)	149 (25.5%)	91 (11.9%)
**Poverty income ratio**^**c**^**, ≥1**	2264 (88.8%)	875 (91.2%)	478 (81.7%)	572 (74.6%)
**Alcohol use (drinks/day)**^**d**^				
**Non-drinkers**	624 (24.5%)	253 (26.4%)	125 (21.4%)	133 (17.3%)
**1–3 drinks/day**	751 (30.5%)	297 (31.0%)	178 (30.4%)	198 (25.8%)
**≥ 4 drinks/day**	336 (14.0%)	121 (12.6%)	54 (9.23%)	139 (18.1%)
**Moderate-to-intense activity**^**e**^**, yes**	1909 (76.6%)	759 (79.1%)	405 (69.2%)	479 (62.5%)
**Calorie intake (kcal)**^**f**^	2174 (21.2)	2210 (31.0)	2163 (40.4)	2145 (38.5)
**Protein intake (g)**^**g**^	86.1 (1.04)	87.3 (1.50)	81.3 (1.93)	87.0 (2.02)
**Body mass index (kg/m**^**2**^**)**^**h**^	27.9 (0.15)	27.7 (0.21)	30.0 (0.30)	29.0 (0.26)
**Body height (cm)**^**i**^	169.0 (0.24)	171.2 (0.33)	170.0 (0.40)	163.2 (0.39)
**Waist circumference (cm)**^**j**^	95.1 (0.36)	95.6 (0.52)	97.5 (0.71)	96.0 (0.62)
**Urinary creatinine (mg/dL)**^**k**^ median (interquartile)	109.3 (55.9, 167.8)	102.0 (50.8, 161.7)	152.1 (99.1, 225.8)	114.6 (66.2, 167.1)
**Serum cotinine (ng/mL)**^**l**^ median (interquartile)	0.03 (0.01, 0.07)	0.03 (0.01, 0.06)	0.03 (0.03, 0.23)	0.03 (0.01, 0.04)
**Serum C-reactive protein (ng/mL)**^**m**^				
**Tertile 1**	373 (14.8)	136 (14.2%)	75 (12.8%)	91 (11.9%)
**Tertile 2**	667 (25.8)	281 (29.3%)	124 (21.2%)	202 (26.3%)
**Tertile 3**	632 (22.3)	230 (24.0%)	149 (25.5%)	216 (28.2%)
**DXA measurement**				
**Total fat mass (kg)**	27.0 (0.27)	27.1 (0.39)	29.4 (0.56)	27.2 (0.45)
**Total fat mass percent**	33.2 (0.20)	32.8 (0.29)	33.5 (0.41)	34.6 (0.35)
**Trunk fat mass (kg)**	12.9 (0.15)	13.1 (0.22)	13.4 (0.28)	13.4 (0.24)
**Trunk fat mass percent**	32.1 (0.21)	31.7 (0.30)	32.4 (0.42)	34.0 (0.34)
**Leg fat mass (kg)**	9.72 (0.10)	9.73 (0.14)	11.4 (0.22)	9.45 (0.17)
**Leg fat mass percent**	35.6 (0.24)	35.1 (0.35)	36.2 (0.45)	36.8 (0.42)
**Trunk/leg fat ratio**	1.36 (0.01)	1.37 (0.02)	1.20 (0.02)	1.45 (0.02)
**Total bone mineral density (g/cm**^**2**^**)**	1.14 (0.003)	1.14 (0.004)	1.20 (0.006)	1.11 (0.004)
**Total lean mass excluding total bone mineral density (kg)**	50.8 (0.31)	52.1 (0.45)	54.4 (0.56)	48.2 (0.51)
**PAHs**^**n**^ (geometric mean [standard error])				
**1-naphthalene (ng/L)**	1254.7 (40.9)	1173.3 (56.5)	1954.6 (107.4)	1188.8 (63.1)
**2-naphthalene (ng/L)**	2848.4 (80.0)	2280.3 (93.0)	4899.1 (218.2)	4564.3 (211.4)
**2-fluorene (ng/L)**	176.7 (4.37)	166.9 (6.13)	283.4 (11.7)	185.3 (6.83)
**3-fluorene (ng/L)**	68.7 (1.80)	64.3 (2.50)	122.6 (5.72)	67.5 (2.66)
**9-fluorene (ng/L)**	236.8 (9.16)	232.4 (13.2)	341.4 (19.3)	223.9 (14.8)
**1-phenanthrene (ng/L)**	112.2 (2.68)	114.4 (4.06)	135.6 (5.31)	106.7 (4.06)
**2-phenanthrene (ng/L)**	52.1 (1.68)	49.7 (2.30)	67.4 (3.53)	55.9 (2.75)
**3-phenanthrene (ng/L)**	75.5 (2.41)	73.9 (3.33)	109.4 (5.75)	68.5 (6.84)
**4-phenanthrene (ng/L)**	21.9 (0.80)	21.0 (1.11)	28.1 (1.56)	22.6 (1.44)
**1-pyrene (ng/L)**	77.7 (1.94)	69.9 (2.60)	103.8 (4.58)	93.8 (3.61)

*Abbreviation*: *DXA* dual-energy x-ray absorptiometry, *PAH* Polycyclic aromatic hydrocarbon

^a^For continuous variables with normal distribution, values are presented as weighted mean (standard error). For continuous variables with skewed distribution, values are presented as geometric mean (standard error) or median (interquartile). For categorical variables, crude numbers and weighted percentages are presented

^b^Unweighted number of participants

^c^177 missing values were replaced by the median values of 2.63

^d^980 missing values

^e^1 missing value

^f^102 missing values were replaced by the median value of 1973 kcal/day

^g^102 missing values were replaced by the median value of 77.2 g/day

^h^13 missing values were replaced by the median value of 27.3 kg/m^2^

^i^12 missing values were replaced by the median value of 166.7 cm

^j^22 missing values were replaced by the median value of 93.6 cm

^k^3 missing values were replaced by the median value of 114.0 mg/dL

^l^302 missing values were replaced by the median value of 0.031 ng/mL

^m^1019 missing values

^n^Based on non-missing participants only: 1-naphthalene, *n* = 2663; 2-naphthalene, *n* = 2673; 2-fluorene; *n* = 2672; 3-fluorene; *n* = 2665; 9-fluorene; *n* = 1187; 1-phenanthrene; *n* = 2676; 2-phenanthrene; *n* = 1663; 3-phenanthrene; *n* = 1670; 4-phenanthrene; *n* = 1132; and 1-pyrene; *n* = 2670

The adjusted correlation coefficients between the log-transformed PAHs ranged between 0.40 and 0.92 (Supplemental Table S1). The adjusted correlations of PAHs in the same class were comparable with those for PAHs in different classes. Fluorene metabolites had higher correlations with phenanthrene metabolites and pyrene metabolites compared with naphthalene metabolites.

Four out of ten PAHs were significantly correlated with total FM% after the FDR correction: 1-naphthalene, 3-fluorene, and 1-pyrene were inversely correlated with higher total FM% (adjusted *r* ranged from − 0.06 to − 0.07) and 4-phenanthrene was positively correlated with total FM% (adjusted *r* = 0.09; all FDR *P*s < 0.05) (Table 2). In addition, six out of ten PAHs were significantly correlated with trunk FM% (Table 2). While 1-naphthalene, 3-fluorene, and 1-pyrene were negatively correlated with trunk FM% (adjusted *r* ranged from − 0.06 to − 0.08), 2-naphthalene, 9-fluorene, and 4-phenanthrene were positively correlated with trunk FM% (adjusted *r* ranged from 0.07 to 0.11; all FDR *P*s < 0.05). These PAHs were generally correlated with the trunk/leg ratio, BMI, and WC, but were not correlated with leg FM%, bone mineral density and total lean mass. Five out of six correlation coefficients of PAHs that were significantly correlated with trunk FM% were statistically stronger than correlation coefficients for leg FM%. Total naphthalene metabolites, total phenanthrene metabolites, total phenanthrene metabolites, and total PAHs were not correlated with body fat distribution.

**Table 2:** Partial Pearson^a^ correlation between creatinine-adjusted PAHs and body fat percentages among non-smokers aged 20 years and older

Pollutant	N	Total fat (%)	Trunk fat (%)	Leg fat (%)	Trunk/leg ratio	Total lean mass (%)	Bone mineral density (g/cm^**2**^)	BMI (kg/m^**2**^)	WC (cm)
**Total naphthalene (ng/g)**	2682	0.0001	0.02	−0.03	0.07	0.001	− 0.06	0.02	0.02
**1-naphthalene (ng/g)**	2663	−0.07^b^	− 0.07^b^	− 0.05	− 0.04	0.05	− 0.07^b^	− 0.10^b^	− 0.10^b^
**2-naphthalene (ng/g)**	2673	0.04	0.07^b,c^	− 0.01	0.11^b^	− 0.03	− 0.04	0.09^b^	0.09^b^
**Total fluorene (ng/g)**	2676	− 0.01	−0.01	− 0.01	− 0.004	0.003	0.03	− 0.03	− 0.04
**2-fluorene (ng/g)**	2672	−0.01	− 0.02	0.01	− 0.04	− 0.003	−0.01	− 0.03	−0.04
**3-fluorene (ng/g)**	2665	−0.07^b^	−0.08^b,c^	− 0.04	−0.07^b^	0.06^b^	−0.03	− 0.09^b^	−0.10^b^
**9-fluorene (ng/g)**	1187	0.08	0.09^b,c^	0.03	0.10^b^	−0.05	0.08	0.08	0.08^b^
**Total phenanthrene (ng/g)**	2682	0.01	0.01	0.0001	0.01	−0.02	0.02	−0.04	− 0.04
**1-phenanthrene (ng/g)**	2676	0.02	0.01	0.02	0.01	−0.02	−0.03	− 0.03	−0.02
**2-phenanthrene (ng/g)**	1663	0.05	0.05	0.03	0.06	−0.05	− 0.04	0.05	0.05
**3-phenanthrene (ng/g)**	1670	−0.04	−0.05	− 0.02	−0.04	0.04	−0.01	− 0.06	−0.05
**4-phenanthrene (ng/g)**	1132	0.09^b^	0.11^b,c^	0.03	0.16^b^	−0.06	0.04	0.07	0.10^b^
**1-pyrene (ng/g)**	2670	−0.06^b^	−0.07^b,c^	− 0.03	−0.04	0.07^b^	−0.08^b^	− 0.04	−0.03
**Total PAHs**	2691	0.03	0.02	0.02	−0.004	−0.03	− 0.03	0.01	− 0.004

*Abbreviation*: *DXA* dual-energy x-ray absorptiometry, *PAH* Polycyclic aromatic hydrocarbon

^a^All correlating variables were log-transformed (e base). Pearson correlation coefficients were adjusted for gender, age (continuous), race/ethnicity (non-Hispanic White, non-Hispanic Black, Hispanic, or others), education (high school or below, any college, and college graduate or above), poverty ratio (< vs. ≥1), moderate-to-vigorous physical activity (yes, no), alcohol use (non-drinkers, 1–3 drinks/day, ≥4 drinks/day), total calorie intake (continuous), protein intake (continuous), serum cotinine levels (continuous), and serum C-reactive protein levels (tertiles). All PAHs accounted for urinary creatinine levels

^b^Significant correlations were adjusted using False Discovery Rate Benjamini-Hochberg Procedure

^c^Statistical difference comparing correlations to leg fat

**Fig. 1:**
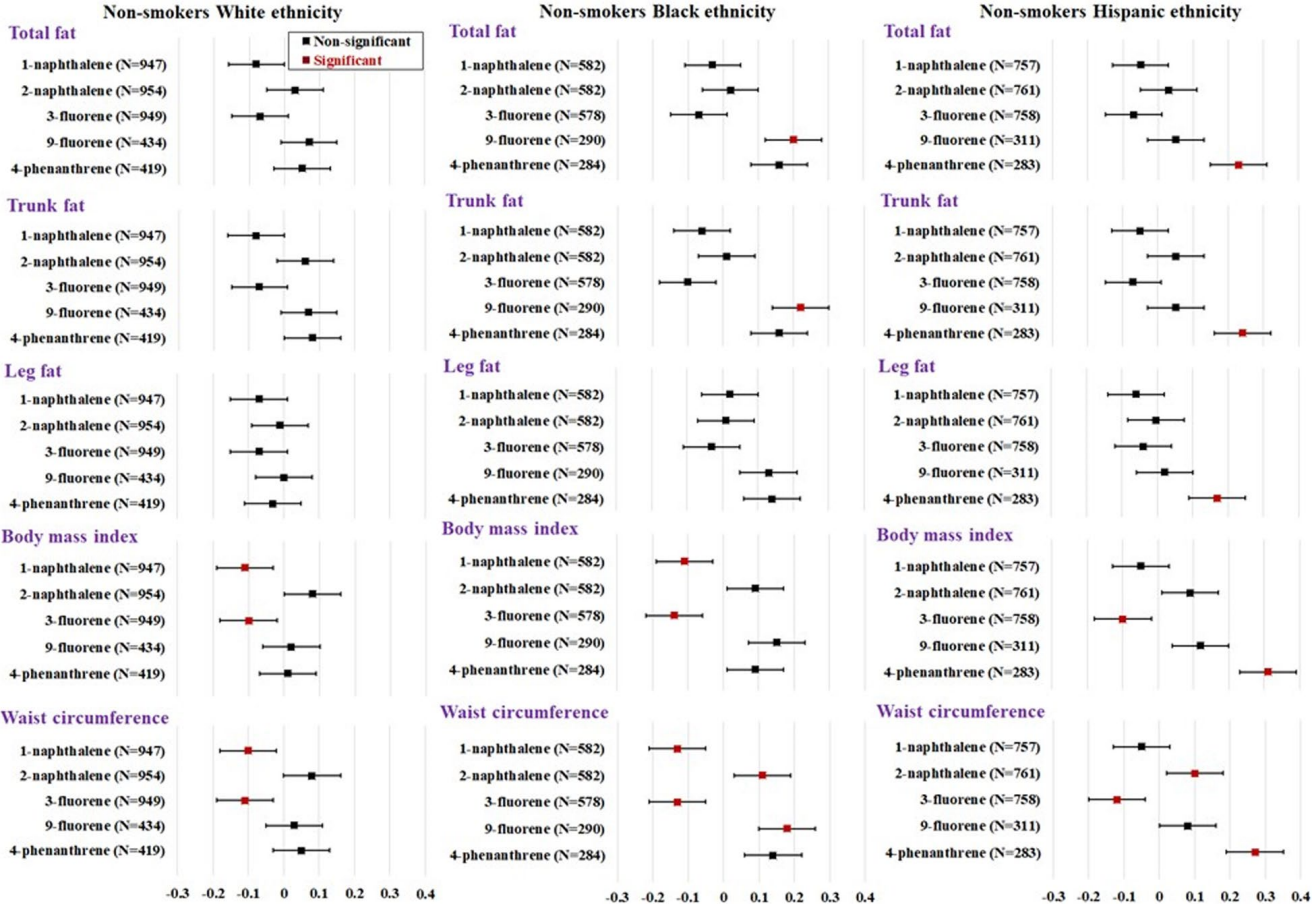
Correlations between selected PAHs that were significantly associated with body fat distribution stratified by race/ethnicity among non-smokers

The strength of PAH-FM% associations was statistically significantly different among three racial/ethnic groups (Tables 3, 4 and 5 & Fig. 1). Specifically, among non-Hispanic Whites, no PAH was correlated with total FM%, trunk FM%, or leg FM% (Table 3). Among non-Hispanic Blacks, 9-fluorene was significantly correlated with higher total FM% (adjusted *r* = 0.20) and trunk FM% (adjusted *r* = 0.22; both FDR *P*s < 0.05) (Table 4). Among Hispanics, 4-phenanthrene was significantly correlated with higher total FM% (adjusted *r* = 0.23) and trunk FM% (adjusted *r* = 0.24; both FDR *P*s < 0.05) (Table 5). The concentrations (geometric mean [standard error]) of 9-fluorene and 4-phenanthrene were higher among non-Hispanic Blacks (341.4 [19.3] ng/L and 28.1 [1.56] ng/L) compared with Hispanics (223.9 [14.8] ng/L and 22.6 [1.44] ng/L) and non-Hispanic Whites (232.4 [13.2] ng/L and 21.0 [1.11] ng/L) (Table 1). When compared across racial/ethnic groups, the correlations of 9-fluorene and 4-phenanthrene with total FM% and trunk FM% were statistically significantly stronger among non-Hispanic Blacks and Hispanics than those of non-Hispanic Whites (*P* for interaction ranged from 0.010 to 0.025) (Tables 3, 4 and 5 & Fig. 1).

**Table 3:** Partial Pearson^a^ correlation between creatinine-adjusted PAHs and body fat percentages among non-smokers with non-Hispanic White ethnicity aged 20 years and older

Pollutant	N	Total fat (%)	Trunk fat (%)	Leg fat (%)	Trunk/leg ratio	Total lean mass (%)	Bone mineral density (g/cm^**2**^)	BMI (kg/m^**2**^)	WC (cm)
**Total naphthalene (ng/g)**	957	−0.01	0.002	− 0.05	0.07	0.01	−0.05	0.01	0.01
**1-naphthalene (ng/g)**	947	−0.08	− 0.08	− 0.07	− 0.03	0.06	−0.10^b^	− 0.11^b^	− 0.10^b^
**2-naphthalene (ng/g)**	954	0.03	0.06^c^	−0.01	0.12^b^	−0.02	0.003	0.08	0.08
**Total fluorene (ng/g)**	952	−0.03	− 0.02	− 0.03	0.01	0.02	0.02	−0.04	− 0.05
**2-fluorene (ng/g)**	950	−0.01	−0.02	0.003	−0.03	0.01	−0.02	− 0.04	− 0.04
**3-fluorene (ng/g)**	949	−0.07	−0.07	− 0.04	−0.06	0.07	−0.05	− 0.10^b^	−0.11^b^
**9-fluorene (ng/g)**	434	0.05	0.07	0.0001	0.11	−0.01	0.08	0.02	0.03
**Total phenanthrene (ng/g)**	953	0.002	0.01	−0.01	0.04	−0.01	− 0.02	−0.03	− 0.02
**1-phenanthrene (ng/g)**	952	0.02	0.02	0.02	0.03	−0.02	−0.04	− 0.01	0.002
**2-phenanthrene (ng/g)**	671	0.04	0.05	0.004	0.07	−0.03	− 0.02	0.05	0.05
**3-phenanthrene (ng/g)**	671	−0.04	−0.04^c^	− 0.04	−0.01^b^	0.04	−0.02	− 0.07	−0.05
**4-phenanthrene (ng/g)**	419	0.05	0.08	−0.03	0.17	−0.01	0.05	0.01	0.05
**1-pyrene (ng/g)**	949	−0.06	− 0.07	−0.03	− 0.05	0.08	− 0.03	−0.02	− 0.01
**Total PAHs**	959	0.03	0.03	0.02	−0.01	−0.04	− 0.04	0.01	− 0.004

*Abbreviation*: *DXA* dual-energy x-ray absorptiometry, *PAH* Polycyclic aromatic hydrocarbon

^a^All correlating variables were log-transformed (e base). Pearson correlation coefficients were adjusted for gender, age (continuous), education (high school or below, any college, and college graduate or above), poverty ratio (< vs. ≥1), moderate-to-vigorous physical activity (yes, no), alcohol use (non-drinkers, 1–3 drinks/day, ≥4 drinks/day), total calorie intake (continuous), protein intake (continuous), serum cotinine levels (continuous), and serum C-reactive protein levels (tertiles). All PAHs accounted for urinary creatinine levels

^b^Significant correlations were adjusted using False Discovery Rate Benjamini-Hochberg Procedure

^c^Statistical difference comparing correlations to leg fat

**Table 4:** Partial Pearson^a^ correlation between creatinine-adjusted PAHs and body fat percentages among non-smokers with non-Hispanic Black ethnicity aged 20 years and older

Pollutant	N	Total fat (%)	Trunk fat (%)	Leg fat (%)	Trunk/leg ratio	Total lean mass (%)	Bone mineral density (g/cm^**2**^)	BMI (kg/m^**2**^)	WC (cm)
**Total naphthalene (ng/g)**	584	0.01	−0.01	0.03	− 0.04	− 0.001	− 0.11	0.02	0.02
**1-naphthalene (ng/g)**	582	−0.03	− 0.06	0.02	− 0.13^b^	0.02	− 0.06	− 0.11^b^	− 0.13^b^
**2-naphthalene (ng/g)**	582	0.02	0.01	0.01	0.03	0.01	−0.12	0.09	0.11^b^
**Total fluorene (ng/g)**	582	0.06	0.06	0.05	0.04	− 0.05	0.002	−0.06	− 0.04
**2-fluorene (ng/g)**	580	0.01	0.003	0.02	−0.004	− 0.001	−0.06	− 0.09	−0.07
**3-fluorene (ng/g)**	578	−0.07	−0.10	− 0.03	−0.10	0.09	− 0.10	−0.14^b^	− 0.13^b^
**9-fluorene (ng/g)**	290	0.20^b^	0.22^b,c^	0.13	0.21^b^	−0.19^b^	−0.03	0.15	0.18^b^
**Total phenanthrene (ng/g)**	585	0.06	0.05	0.04	0.02	−0.07	0.05	−0.05	− 0.04
**1-phenanthrene (ng/g)**	584	0.05	0.04	0.04	0.02	−0.04	−0.07	− 0.03	−0.01
**2-phenanthrene (ng/g)**	372	0.11	0.10	0.08	0.06	−0.09	− 0.13	0.06	0.07
**3-phenanthrene (ng/g)**	376	−0.01	−0.03	0.003	−0.04	0.02	−0.07	− 0.08	−0.06
**4-phenanthrene (ng/g)**	284	0.16	0.16	0.13	0.14	−0.11	− 0.03	0.09	0.14
**1-pyrene (ng/g)**	581	−0.02	−0.03	0.01	−0.02	0.04	−0.12^b^	− 0.05	−0.03
**Total PAHs**	585	0.02	0.01	0.03	−0.02	−0.03	− 0.004	−0.01	− 0.01

*Abbreviation*: *DXA* dual-energy x-ray absorptiometry, *PAH* Polycyclic aromatic hydrocarbon

^a^All correlating variables were log-transformed (e base). Pearson correlation coefficients were adjusted for gender, age (continuous), education (high school or below, any college, and college graduate or above), poverty ratio (< vs. ≥1), moderate-to-vigorous physical activity (yes, no), alcohol use (non-drinkers, 1–3 drinks/day, ≥4 drinks/day), total calorie intake (continuous), protein intake (continuous), serum cotinine levels (continuous), and serum C-reactive protein levels (tertiles). All PAHs accounted for urinary creatinine levels

^b^Significant correlations were adjusted using False Discovery Rate Benjamini-Hochberg Procedure

^c^Statistical difference comparing correlations to leg fat

**Table 5:** Partial Pearson^a^ correlation between creatinine-adjusted PAHs and body fat percentages among non-smokers with Hispanic ethnicity aged 20 years and older

Pollutant	N	Total fat (%)	Trunk fat (%)	Leg fat (%)	Trunk/leg ratio	Total lean mass (%)	Bone mineral density (g/cm^**2**^)	BMI (kg/m^**2**^)	WC (cm)
**Total naphthalene (ng/g)**	763	−0.002	0.02	−0.03	0.10	0.01	−0.07	0.04	0.06
**1-naphthalene (ng/g)**	757	−0.05	−0.05	− 0.06	0.01	0.03	−0.04	− 0.05	−0.05
**2-naphthalene (ng/g)**	761	0.03	0.05	−0.004	0.11	0.004	−0.06	0.09	0.10^b^
**Total fluorene (ng/g)**	762	0.01	0.02	0.004	0.03	−0.04	−0.06	−0.03	− 0.06
**2-fluorene (ng/g)**	762	0.02	0.02	0.03	0.01	−0.05	−0.14^b^	− 0.02	−0.05
**3-fluorene (ng/g)**	758	−0.07	−0.07	− 0.04	−0.04	0.06	−0.13^b^	− 0.10^b^	−0.12^b^
**9-fluorene (ng/g)**	311	0.05	0.05	0.02	0.04	−0.05	− 0.02	0.12	0.08
**Total phenanthrene (ng/g)**	764	0.03	0.04	0.01	0.04	−0.06	−0.04	− 0.03	−0.07
**1-phenanthrene (ng/g)**	762	−0.01	−0.004	− 0.03	0.02	− 0.01	−0.09	− 0.04	−0.07
**2-phenanthrene (ng/g)**	450	0.13	0.13	0.11	0.07	−0.14^b^	−0.10	0.17^b^	0.10
**3-phenanthrene (ng/g)**	453	−0.03	−0.03	− 0.02	−0.04	0.02	−0.12	− 0.03	−0.09
**4-phenanthrene (ng/g)**	283	0.23^b^	0.24^b^	0.17^b^	0.16	−0.20^b^	0.07	0.31^b^	0.27^b^
**1-pyrene (ng/g)**	760	−0.05	−0.05	− 0.04	−0.01	0.05	−0.15	− 0.02	−0.03
**Total PAHs**	767	0.03	0.05	−0.01	0.11	−0.03	−0.07	0.04	0.05

*Abbreviation*: *DXA* dual-energy x-ray absorptiometry, *PAH* Polycyclic aromatic hydrocarbon

^a^All correlating variables were log-transformed (e base). Pearson correlation coefficients were adjusted for gender, age (continuous), education (high school or below, any college, and college graduate or above), poverty ratio (< vs. ≥1), moderate-to-vigorous physical activity (yes, no), alcohol use (non-drinkers, 1–3 drinks/day, ≥4 drinks/day), total calorie intake (continuous), protein intake (continuous), serum cotinine levels (continuous), and serum C-reactive protein levels (tertiles). All PAHs accounted for urinary creatinine levels

^b^Significant correlations were adjusted using False Discovery Rate Benjamini-Hochberg Procedure

## Discussion

In this large sample of US non-smoking adults, we found various correlation patterns between urinary PAH levels and regional body fat distribution. While 1-naphthalene, 3-fluorene, and 1-pyrene were inversely correlated with higher body fat contents, we observed positive correlations for 2-naphthalene, 9-fluorene, and 4-phenanthrene with the same body composition variables. Relevant to exposure disparities, higher PAH concentrations were observed for non-Hispanic Blacks and Hispanics compared to non-Hispanic Whites. Perhaps linked to these higher PAH levels, we found that positive correlations of 9-fluorene and 4-phenanthrene with total FM% and trunk FM% were statistically significantly stronger among non-Hispanic Blacks and Hispanics compared to non-Hispanic Whites. Of PAHs that were associated with body fat, five out of six PAHs had significantly stronger correlations with trunk FM% compared to leg FM%.

To the best of our knowledge, the current analysis is among the first to assess correlations between individual PAHs and body fat distribution among non-smokers, adjusting for a variety of potential confounders. Prior studies in adults evaluating individual PAHs and obesity were scarce [6, 16, 17]. In addition, these studies included both smokers and non-smokers, which can impact the association between PAHs and body fat distribution [24]. Also, previous studies used BMI as the body fatness measure, which could not differentiate between body fat and muscle weight, nor capture visceral fat [34]. In an analysis based on the NHANES 2013–2014 data, total PAHs were not correlated with BMI among adults aged 20–80 years old [16]. A study based on the Korean National Environmental Health Survey found that 2-naphthalene and total PAH were positively correlated with BMI among 3787 adults [17]. In another study among 4765 adults from the 2001–2008 NHANES study, 2-phenanthrene had a monotonic dose-response relationship with higher BMI [6], while 1-naphthalene was inversely correlated with BMI [6]. In the current analysis, we observed the positive correlation of 2-naphthalene and body fat as well as the negative correlation of 1-naphthalene with body fat, but not the positive correlation between 2-phenanthrene and body fat. The differences in our results compared to others might be due to the fact that our study only included non-smokers, where the PAH concentrations tend to be lower and thus the association may differ from the prior study that included smokers [6], who have higher levels of PAHs.

In addition to overall body fat contents, we also examined regional fat distribution. Interestingly, we observed consistently stronger correlations of PAHs with trunk fat compared with leg fat, which may suggest that PAHs impact trunk fat and leg fat differently through differed pathophysiological pathways. Compared with the lower-body fat, the upper-body fat is more active on fat metabolism [35] and has higher exchange of blood flow [36], and thus may favor the storage in and the release of PAHs from fat in this region. Alternatively, the observed differences in the correlations between PAHs and body fat distribution may suggest stronger influences of PAHs on fat metabolism in the trunk area. Similarly, other endocrine-disrupting chemicals have also shown stronger correlations with trunk fat compared with leg fat [32]. In addition to obesity, PAHs are also associated with other cardiometabolic diseases such as diabetes [5, 6], dyslipidemia [6], hypertension [6, 7], and cardiovascular disease [7]. Furthermore, the accumulation of trunk fat, but not leg fat, is associated with increased metabolic diseases [18–20]. Therefore, our results are in line with the notion that PAH exposures may potentially link to cardiometabolic diseases through the accumulation of visceral fat. Nevertheless, more epidemiological and mechanistic studies are needed to further elucidate these findings.

PAHs may influence adiposity through a few pathways. First, PAHs are structurally similar to estrogen and have estrogenic properties [37, 38], and may disrupt estrogen-mediated pathways [10, 11]. Phenanthrene and fluoranthene metabolites have been shown to have antiandrogenic effects [39]. Naphthalene metabolites may act as thyroid hormone receptor antagonists [9]. Furthermore, PAHs could alter dopamine and serotonin signaling, which may subsequently impact eating behaviors [40]. PAHs can also accumulate in tissues with high fat contents and inhibit lipolysis, thus resulting in increased fat mass accumulation and weight gain [12, 41]. It is worth noting that we observed different correlation patterns between individual PAHs and body fat distribution. A previous study suggested that PAHs may have different actions on the estrogen-responsive genes [10], which might explain the observed different impact on body fat distribution in the current study. However, this study did not include individual PAHs that were assessed in the current study, and further studies are needed to elucidate the mechanisms under varying correlations of PAHs on body fat distribution.

Interestingly, we observed racial/ethnic differences in the correlations between PAHs and body fat contents: PAHs, particularly 9-fluorene and 4-phenanthrene, had statistically significantly stronger correlations with total FM% and trunk FM% among non-Hispanic Blacks and Hispanics compared to non-Hispanic Whites. The heterogeneity may be partially explained by the racial/ethnic differences in environmental exposures noted by higher concentrations of PAHs among non-Hispanic blacks compared to non-Hispanic whites. While previous studies have found that racial/ethnic differences in total FM%, trunk FM%, or visceral fat, as well as lower metabolic rate and fat oxidation [42–44] in non-Hispanic Blacks compared to non-Hispanic whites, these differences have not been evaluated in the context of disparate environmental exposures that may be associated with these health outcomes. Indeed, differences in lifestyle factors may drive these racial/ethnic exposure differences. Prior studies suggested that the main exposure to 9-fluorene and 4-phenanthrene was through diet such as grilled/charred meats or foods, contaminated flour, and bread products, processed and pickled foods, and contaminated water and cow’s milk [2, 3, 45]. Moreover, second-hand smoking at home has also shown to be associated with higher levels of fluorene and phenanthrene metabolites [46]. Compared with non-Hispanic Whites, non-Hispanic Blacks tended to consume more meat prepared at higher temperatures (e.g., grilled, charred, fried) [47–49]. In addition, non-Hispanic Blacks and Hispanics are more likely to reside within socioeconomically disadvantaged neighborhoods and thus are disproportionally exposed to higher levels of air pollution (from traffic, factory, construction, cooking, etc.) [50] and secondhand smoke [51] compared to non-Hispanic Whites. These disproportionately higher exposure to sources of PAHs could be further modified by factors such as psychosocial stress [52] that are prevalent among non-Hispanic Black and Hispanic populations with implications on health outcomes linked to adiposity. As such, our findings of racial/ethnic differences might indicate that non-Hispanic Blacks and Hispanics are exposed to higher PAH exposures and may be particularly susceptible to PAH’s obesogenic effects due to higher exposure to PAH sources. It is also likely that there are other race/ethnic-related factors that modulate the associations between these environmental toxicants and obesity-related outcomes, although more research is needed to further elucidate potential mechanisms underlying these racial/ethnic disparities.

Our study was based in a representative population of the US adults. A major strength of our study is the evaluation of associations between PAHs and comprehensive measurements of fat depots, including fat mass and lean mass. We also controlled for a wide range of confounding factors, such as age, gender, diet intake, and serum levels of cotinine and CRP. In addition, we assessed the potential effect modification by race/ethnicity on the associations between PAHs and body fat distribution.

Nevertheless, our study had several limitations. First, the current study used the cross-sectional data, thus the temporal relations cannot be determined. In addition, we could not rule out the possibility that the increased PAH levels may reflect the increased storage and release of PAHs from adipose tissues. Second, the current study did not include measurements of some high-molecular-weight PAHs, such as the well-studied benzo[a]pyrene. High-molecular-weight PAHs were mainly excreted via bile and thus were below the detection limit in the urinary samples [45]. Third, the current study used a single urine sample to measure levels of PAHs; therefore, the long-term exposure of PAHs may not be sufficiently reflected. A prior study showed that measurements of other lipophilic chemicals in a single urine spot test is moderately predictive of long-term exposure over 3 months [53]. Future studies are warranted to replicate the findings using repeated assessments of PAHs in prospective cohort studies to offer potential insights into the temporal relationships between PAHs and body fat distribution.

## Conclusion

In conclusion, various correlation patterns between urinary PAH levels and regional body fat distribution were observed in this large sample of US adult non-smokers. In comparison with non-Hispanic Whites, the positive correlations of 9-fluorene and 4-phenanthrene with total FM% and trunk FM% were statistically significantly stronger among non-Hispanic Blacks and Hispanics who had higher PAH exposures. These results suggest that these racial/ethnic groups might be particularly susceptible to the detrimental effects of PAH exposures and future studies are warranted to further explore the mechanisms underlying the racial/ethnic disparity observed in the current study. Such studies could aid in informing strategies for interventions to reduce exposure sources of PAHs in racial/ethnic disparities that contribute to obesity-related health outcomes.

## Supplementary Information

**Additional file 1: Supplemental Table S1.** Pearson correlation among PAHs among non-smokers. **Supplemental Figure S1.** Flowchart of the study design of the current analysis.

## Data Availability

The data used in the current study is openly accessible in the NHANES website: https://www.cdc.gov/nchs/nhanes/index.htm.
