# Supplementary material for: Urinary polycyclic aromatic hydrocarbon excretion and regional body fat distribution: evidence from the U.S. National Health and Nutrition Examination Survey 2001–2016
Source: Environ Health. 2022 Aug 10;21:75. doi: 10.1186/s12940-022-00890-8 (PMC9364531; doi:10.1186/s12940-022-00890-8)
Supplement: Supplementary file 1 — Additional file 1: Supplemental Table S1. Pearson correlation among PAHs among non-smokers. Supplemental Figure S1. Flowchart of the study design of the current analysis. [file 12940_2022_890_MOESM1_ESM.docx]

**Supplemental Table S1.** Pearson^a^ correlation among PAHs among non-smokers.

|  | **1-naphthalene** | **2-naphthalene** | **2-fluorene** | **3-fluorene** | **9-fluorene** | **1-phenanthrene** | **2-phenanthrene** | **3-phenanthrene** | **4-phenanthrene** | **1-pyrene** |
| --- | --- | --- | --- | --- | --- | --- | --- | --- | --- | --- |
| **1-naphthalene** | 1.00 | - |  | - | - | - | - | - | - | - |
| **2-naphthalene** | 0.40 | 1.00 | - | - | - | - | - | - | - |  |
| **2-fluorene** | 0.58 | 0.54 | 1.00 | - | - | - | - | - | - |  |
| **3-fluorene** | 0.58 | 0.53 | 0.92 | 1.00 | - | - | - | - | - |  |
| **9-fluorene** | 0.51 | 0.54 | 0.79 | 0.74 | 1.00 | - | - | - | - |  |
| **1-phenanthrene** | 0.55 | 0.50 | 0.82 | 0.77 | 0.85 | 1.00 | - | - | - |  |
| **2-phenanthrene** | 0.45 | 0.59 | 0.79 | 0.75 | 0.85 | 0.83 | 1.00 | - | - |  |
| **3-phenanthrene** | 0.53 | 0.51 | 0.81 | 0.81 | 0.82 | 0.83 | 0.79 | 1.00 | - |  |
| **4-phenanthrene** | 0.45 | 0.51 | 0.70 | 0.65 | 0.79 | 0.80 | 0.83 | 0.78 | 1.00 |  |
| **1-pyrene** | 0.40 | 0.60 | 0.64 | 0.65 | 0.72 | 0.67 | 0.77 | 0.70 | 0.69 | 1.00 |

^a^All correlating variables were log-transformed (e base) and adjusted for gender, age (continuous), race/ethnicity (non-Hispanic White, non-Hispanic Black, Hispanic, or others), education (high school or below, any college, and college graduate or above), poverty ratio (< vs. ≥1), moderate-to-vigorous physical activity (yes, no), alcohol use (non-drinkers, 1-3 drinks/day, ≥4 drinks/day), total calorie intake (continuous), protein intake (continuous), serum cotinine levels (continuous), and serum C-reactive protein levels (tertiles).

**Supplemental Figure S1.** Flowchart of the study design of the current analysis.

U.S. residents surveyed between 2001-2002, 2003-2004, 2005-2006,

2011-2012, 2013-2014, and 2015-2016 (n=61,049)

DXA scan completed (n=20,709)

Without medical examination data (n=2,284)

Age less than 20 years (n=27,842)

Adults examined at MEC (n=30,923)

Scan completed, but invalid (n=2)

Not scanned, weight > 300 lbs (n=185)

Not scanned, height > 6’5” (n=9)

Not scanned, other reason (n=10,018)

PAHs measured in random 1/3 participants with blood samples and enriched with smokers (n=17,031)

Participants with both PAHs and DXA data (n=7,531)

Physical, mental, or emotional limitations (n=240)

Self-reported chronic diseases (n=2,264)

Final analyzing sample (n=2,691)

Without smoking status data (n=3)

Smokers (n=2,333)
